# Radiographic comparison of atelocollagen versus deproteinized bovine bone minerals covered with a collagen membrane in alveolar ridge preservation: a retrospective study

**DOI:** 10.1186/s12903-023-03647-y

**Published:** 2023-11-21

**Authors:** Sha You, Fan Yu, Qihang Fan, Ting Xia, Liang Liang, Qi Yan, Hao Zeng, Bin Shi

**Affiliations:** 1 State Key Laboratory of Oral & Maxillofacial Reconstruction and Regeneration, Key Laboratory of Oral Biomedicine Ministry of Education, Hubei Key Laboratory of Stomatology, School & Hospital of Stomatology, Wuhan University, Wuhan, China; 2 Department of Implantology, School & Hospital of Stomatology, Wuhan University, Wuhan, China; 3 Department of Oral Implantology, Jianli Stomatology Hospital, Dongguan, China

**Keywords:** Alveolar ridge preservation, Atelocollagen, Radiographic evaluation, Three-dimensional image

## Abstract

**Background:**

Atelocollagen (AC) is a low-immunogenic collagen derivative with longer degradation time, which can be a suitable material for alveolar ridge preservation (ARP). However, there are few human studies on AC using for ARP. This research aims to radiographically evaluate the efficacy of AC in comparison to deproteinized bovine bone minerals covered with a collagen membrane (DBBM/CM) in ARP.

**Methods:**

Medical records in the Implantology Department of the Hospital of Stomatology of Wuhan University were screened for patients who received flapless ARP using either AC or DBBM/CM. A total of 58 patients were included in this retrospective study. 28 patients were treated with AC, while 30 patients were used DBBM/CM. Cone-beam computed tomography (CBCT) scans were taken before extraction and after 6 months of healing. To assess the dimensional change of the extraction sockets, the scanning data were output and transferred to the digital software to measure horizontal bone width change, vertical bone height change and bone volume change in region of interest. To evaluate the bone quality of healed sockets, the bone density of virtual implants was evaluated.

**Results:**

The horizontal bone width changes at all five different levels showed no significant difference between the two groups. The largest horizontal bone width decrement in both groups occurred at the crest of ridge, which decreased 3.71 ± 1.67 mm in AC group and 3.53 ± 1.51 mm in DBBM/CM group (*p* = 0.68). At the central buccal aspect, the ridge height reduced 0.10 ± 1.30 mm in AC group, while increased 0.77 ± 2.43 mm in DBBM/CM group (*p* = 0.10). The vertical bone height differences between two groups showed no statistical significance. The percentages of volume absorption in AC group and DBBM/CM group were 12.37%±6.09% and 14.54%±11.21%, respectively. No significant difference in volume absorption was found (*p* = 0.36). The average bone density around virtual implants in AC group (649.41 ± 184.71 HU) was significantly lower than that in DBBM/CM group (985.23 ± 207.85 HU) (*p* < 0.001).

**Conclusions:**

ARP with AC had a similar effect on limiting the dimensional alteration of alveolar ridge, when radiographically compared with DBBM/CM.

**Supplementary Information:**

The online version contains supplementary material available at 10.1186/s12903-023-03647-y.

## Background

Alveolar ridge dimensional reduction after tooth extraction could have negative effect on treatment for restoring missing teeth, such as dental implant restorations [1]. Alveolar ridge preservation (ARP) after tooth extraction has been proposed to maintain the alveolar ridge dimension and simplify the subsequent treatment [2]. Shreds of literature have confirmed that ARP can reduce alveolar ridge resorption compared to natural healing after tooth extraction [3–5].

Collagen material has been used in dental clinics for the past decades, due to its highly porous structure, good biocompatibility, and biodegradability. Collagen can help to stabilize the blood colt which is critical to induce the regeneration process in the wound. Besides, collagen provides a favorable environment for the osteoblast-like cells attachment, proliferation, and differentiation [6]. These advantages make collagen a potential alternative material for ARP. Collagen materials were combined with bone graft materials to fill extraction sockets or used as a sealing material in ARP [7]. However, using collagen plug alone in ARP is still controversial in clinical practice, since it may have negative effect on ARP [8]. One of the possible reasons for the compromised outcome of collagen plug in ARP is the weak space maintenance ability, resulting from its rapid degradability. Another possible reason is the allergic reactions of the host body, which can induce harmful inflammation to interfere the healing process. Although collagen has always been regarded as a weak antigen [9], its allergic reactions are still occasionally reported. Clinical observations indicated that 2 ~ 4% of the total population possess an inherent immunity (allergy) to bovine type I collagen [10, 11]. Therefore, collagen derivative with prolonged degradation time and decreased immunogenicity may have the potential to facilitate the healing of extraction socket and maintaining the alveolar ridge dimension.

Atelocollagen (AC) is a low-immunogenic collagen derivative that removed the N- and C-terminal telopeptide components of collagen molecules, which are the major antigenic determinants for certain donor/recipient pairings [12, 13]. Treatment with AC did not induce the toxicity related gene expression level, suggesting it to be a potential non-toxic candidate for wound healing [14]. Besides, AC collects to form a fiber-like natural collagen under physiological conditions. This means AC can exist in living body for a long time, which may prolong the time of space maintenance [15]. In addition, AC can be made from type I collagen, which is an important matrix of nature bone and can facilitate the osteogenic differentiation of mesenchymal stem cells. AC can also be produced as a plug form, which can be used easily. Thus, AC can be a suitable material for ARP. In past decades, AC has been used to fill the extraction socket of third molar to promote healing and minimize complications [16]. Recently, an animal study found ARP with AC did not disturb healing of the extraction socket [17]. However, there are few human studies on AC using for ARP, the efficiency of AC is still needing to be investigated. Since strong lines of evidence from preclinical and clinical studies have confirmed the effectiveness of deproteinized bovine bone minerals covered with a collagen membrane (DBBM/CM) in ARP [18–20], it is strongly recommended to fill the extraction socket with bone graft materials and to seal it with an autologous or exogenous barrier [21]. Therefore, the ARP with DBBM/CM was selected as a positive control in this study. The aim of this retrospective study is to radiographically evaluate the efficacy of AC in comparison to DBBM/CM in ARP.

## Methods

### Study design

This was a retrospective cohort study to compare the radiographic evaluation between AC and DBBM/CM in ARP. The primary outcomes were horizontal width change of extraction socket. Secondary outcomes included vertical height change, bone volume change and bone density measurement.

The study protocol was approved by the Ethics Committee of the Hospital of Stomatology, Wuhan University (NO.2022B56). Informed consents were obtained from patients. The present study was performed in compliance with the Helsinki Declaration and the Strengthening the Reporting of Observational Studies in Epidemiology (STROBE) checklist.

### Study population

All patients who received ARP using either AC or DBBM/AC conducted from May 2019 to May 2022 in the Implantology Department of Hospital of Stomatology, Wuhan University were screened. The clinical and radiographic workup documented in the electronic health record and intraoperative photographs were checked to assist with screening and enrollment of subjects. A total of 58 patients were included in the light of the following inclusion criteria: a) ≥ 18 years of age; b) the extraction socket had a radiographic buccal bone wall width < 1 mm or existed a slight buccal bone defect (buccal bone loss < 50%) or had a chronic infection around the socket; c) no uncontrolled systemic diseases or presenting a contraindication to extraction; d) good oral hygiene. The exclusion criteria were as follow: (a) flap surgery; (b) history of radiotherapy and chemotherapy in the past 5 years; (c) sites with acute inflammation; (d) heavy smokers (> 10 cigarettes per day); (e) females in pregnancy or lactation; (f) bone diseases such as cysts or tumors; (g) history of intravenous bisphosphonate.

### Calculation of sample size

In the previous study [22], the standard deviation of horizontal ridge width in the DBBM/CM group was 1.35 mm. The estimate of the standard deviation in the AC group was 1.18 mm in the pilot study. A 1.0 mm difference was suggested. Two-sided significance level was set to 0.05 and the desired power level to 80%. According to the data, a least sample size of 27 per group was determined by PASS software version 15 (NCSS, LLC. Kaysville, UT, USA). 28 patients using AC and 30 patients using DBBM/CM were eventually included.

### Surgical procedures

**Fig. 1:**
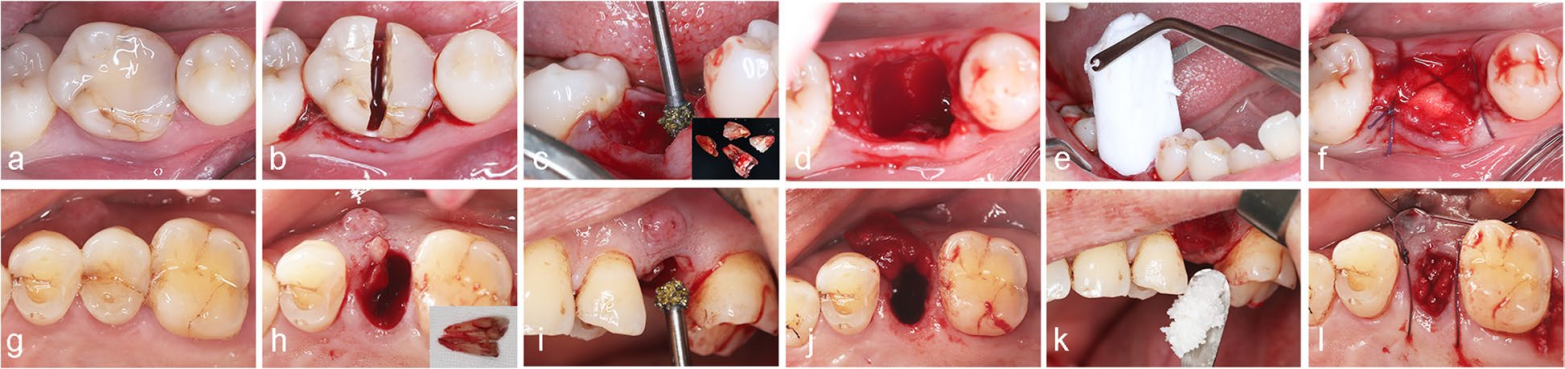
Surgical procedures of the AC group and DBBM/CM group. **a**-**f** Surgical procedures of using atelocollagen in ARP. **g**-**l** Surgical procedures of using DBBM/CM in ARP.

All patients were performed by two experienced dentists (B. Shi and H. Zeng). After local anesthesia (2% lidocaine with 1:100000 epinephrine) and disinfection of the surgical area, the tooth was removed atraumatically without harming the bony wall by experienced doctors with flapless procedure. The extraction sockets were cleaned carefully with surgical curettes and irrigated by using saline solution. Then, in the test group, an AC plug (Teruplug, Olympus Terumo Biomaterials, Japan) was applied to fill the socket (Fig. 1a-f). While in the control group, the socket was filled with DBBM (Bio-Oss, Geistlich Pharma AG, Switzerland) /CM (Bio-Gide, Geistlich Pharma AG, Switzerland) (Fig. 1g-l). Both groups received the crossed mattress suture to fix the materials. Patients were instructed to rinse with 0.2% chlorhexidine digluconate once a day for at least 2 weeks. Smokers were recommended to quit smoking, especially for first 2 weeks after surgery. Non-steroidal anti-inflammatory drugs were recommended for one week. Analgesics were used only when necessary. Sutures were removed 14 days after surgery.

### Radiographic measurements and analysis

All the patients underwent cone beam computed tomography (CBCT) scanning before ARP (T0) and at 6 months after ARP (T1). The scanning data were output and transferred to a three-dimensional (3D) image generation and editing software (Mimics 21.0, Materialise, Leuven, Belgium) to measure the horizontal width and vertical height. For further evaluation, another 3D volume visualization software (3-matic 13.0, Materialise, Leuven, Belgium) was employed to analyze volumetric changes between the AC group and DBBM/CM group. The bone density at T1 was measured by implant design software. The above-mentioned radiographic measurements were performed by two independent examiners (S. You and F. Yu) who were not involved in the surgical procedure and received prior training.

**Fig. 2:**
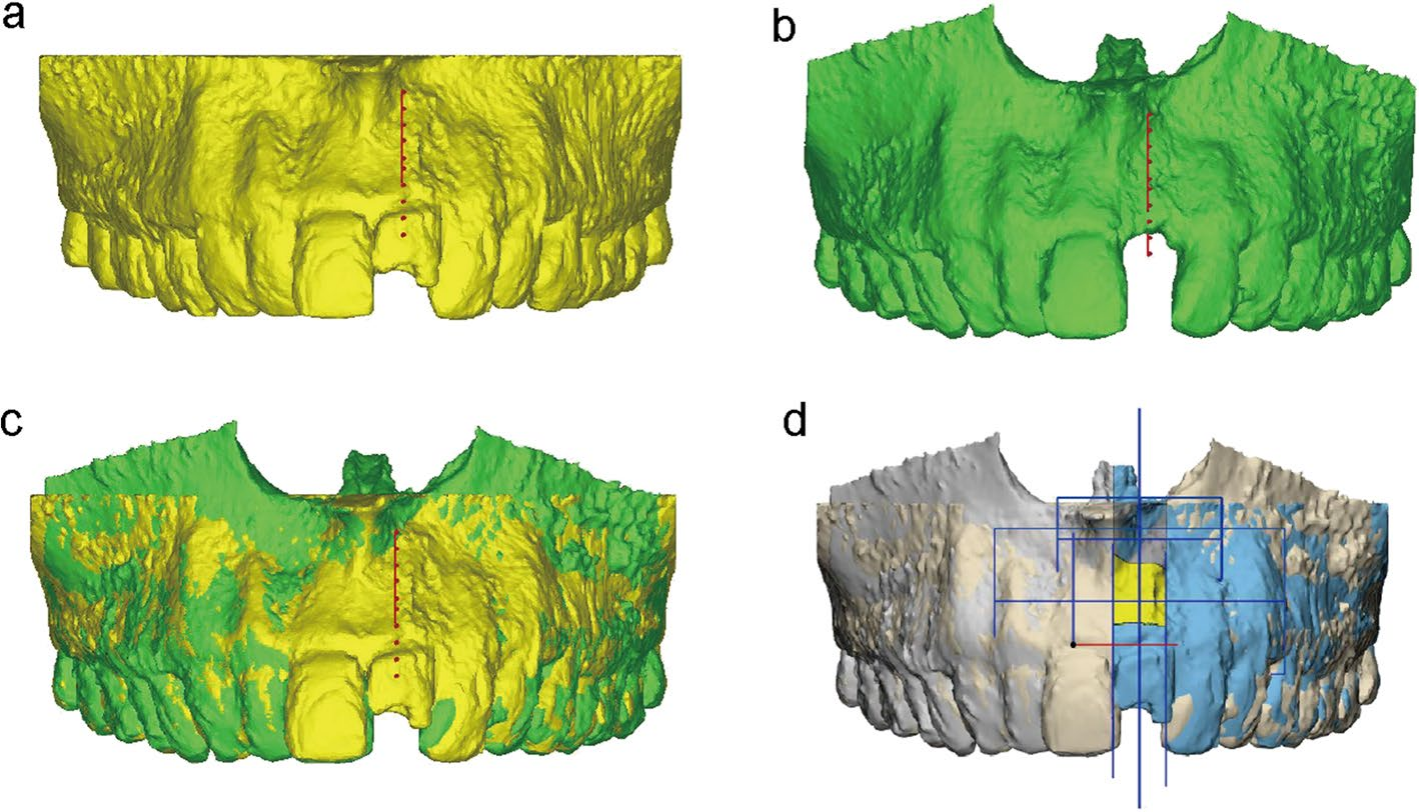
**a** 3D virtual reconstruction of CBCT data at T0. **b** 3D virtual reconstruction of CBCT data at T1. **c** Superimposition of virtual models at T0 and T1. **d** ROI (yellow) in the superimposed model

**Fig. 3:**
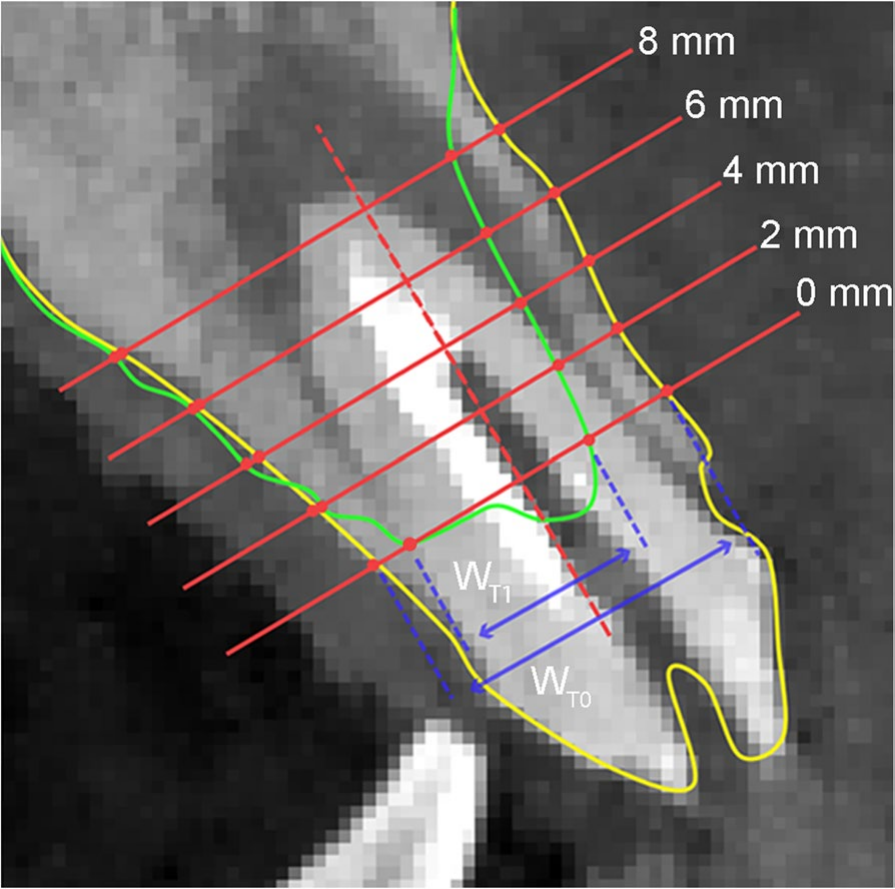
The diagram illustrating the landmarks used for measurement. Yellow line: hard tissue profile at T0; Green line: hard tissue profile at T1. The dotted red line was drawn as a reference line. Four lines perpendicular to the reference line at 0, 2, 4, 6, 8 mm below the baseline of the ridge crest at T1 were intersected with two hard tissue profile. Bone width at T0 and T1was denoted as W_T0_ and W_T1_, respectively

Based on the unique points and surfaces of the remaining teeth, the superposition and aligning of two scanning images (CBCT taken on T0 and T1) were conveniently obtained and explicitly displayed (Fig. 2a, b and c). A center plane between teeth adjacent to the target position and its long axis were used to determine the measurement plane. Then, 5 parallel lines perpendicular to long axis at 0, 2, 4, 6, 8 mm below the ‘baseline’ of the collapsed alveolar ridge after 6 months of healing were drawn to measure horizontal width changes at different levels [23–26] (Fig. 3). The horizontal bone width changes were figured out on the basis of ΔT0-T1.

The distances from buccal and lingual alveolar crest (before and after extraction) to the collapsed ‘baseline’ were measured (above the ‘baseline’ was positive, while below the ‘baseline’ was negative). The vertical height decreases were figured out based on ΔT0-T1.

In accordance with the buccal, lingual and approximal alveolar crest, vertical levels of 0 and 8 mm, the region of interest (ROI) was gained by converting STL to CAD files with 3-matic software for 3D measurement and analysis (Fig. 2d). The bone loss was determined with ΔT0-T1.

To evaluate the bone density of healed socket, 3Shape Implant Studio software version 1.7.18.1 (3Shape A/S, Copenhagen, Denmark) was employed to virtually place implants at healed socket site by using CBCT data at T1 and perform the bone density visualization around planned implants, which visually described bone density classification around its surface. Meanwhile, the measurement of bone density of virtual implants was carried out by Simplant Pro software version 18.0 (Dentsply Implants NV, Hasselt, Belgium), which was determined as mean HU values [27]. Density analyzed sample number of each virtual implant was set as 150 and analysis shell thickness was set as 1 mm by the software algorithm.

### Statistical analysis

To minimize the measurement bias, two well-trained observers took all measurement independently. The average of the values ​​measured by two observers was used as the final values. Statistical analyses were conducted using SPSS software version 19.0 (IBM company, Armonk, NY, USA). The data were reported by means ± standard deviations (SD). Two independent sample t-test or Mann-Whitney U test was selected to evaluate changes between groups. *P* ≤ 0.05 was considered statistically significant.

## Results

### Patient demographics

A total of 58 patients were included in the present study. In AC group, the mean age was 41.6 ± 16.0years, 17 women and 11men. In DBBM/CM group, the mean age was 39.7 ± 14.7 years, 12 women and 18 men. The demographic data of the patients are presented in Table 1. The AC group consisted of 9 anterior teeth and 19 posterior teeth, the reasons for extraction were divided into endodontic complications (*n* = 14) or periodontally compromised (*n* = 14). The DBBM/CM group consisted of 16 anterior teeth and 14 posterior teeth, the reasons for extraction were endodontic complications (*n* = 19) or periodontally compromised (*n* = 11). All the ARP surgeries were successfully performed, and no intra-operative complications were recorded.

**Table 1:** Demographic data of the enrolled patients with descriptive variables

	AC (*N* = 28)	DBBM/CM (*N* = 30)
**Gender**	Male: 11	Male: 18
	Female: 17	Female: 12
**Age (mean ± SD, Yrs)**	41.57±16.02	39.70±14.73
**Reason of extraction**	Endodontics: 14	Endodontics: 19
	Periodontitis: 14	Periodontitis: 11
**Location**	Anterior tooth: 9	Anterior tooth: 16
	Posterior tooth: 19	Posterior tooth: 14
**Chronic inflammation**	None: 6	None: 7
	Exist: 22	Exist: 23
**Socket integrity**	Complete: 14 Incomplete: 14	Complete: 11
		Incomplete: 19

### Horizontal ridge width changes

**Fig. 4:**
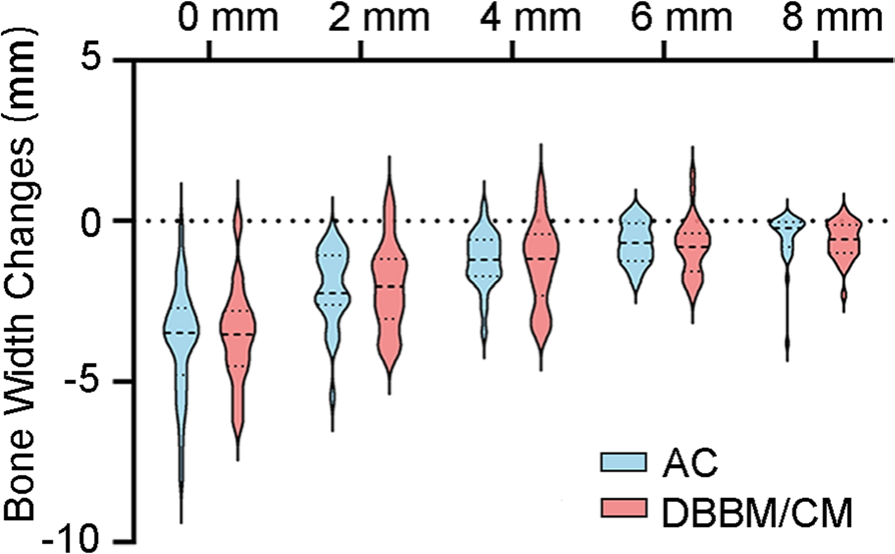
Measurement of horizontal bone width changes at different levels in the AC group and DBBM/CM group

The horizontal bone alteration parameters and the percentages at five different vertical levels are summarized in Table 2; Fig. 4. At the level of 0 mm, the mean bone loss was 3.71 ± 1.67 mm in AC group and 3.53 ± 1.51 mm in DBBM/CM group (*p = 0.68*). At 2 mm level, the mean bone loss was 2.08 ± 1.17 mm and 2.04 ± 1.34 mm, respectively (*p* = 0.91). At 4 mm level, the parameter was 1.21 ± 0.85 mm and 1.33 ± 1.27 mm, respectively (*p* = 0.68). At 6 mm level, the mean bone loss was 0.69 ± 0.64 mm and 0.79 ± 0.87 mm, respectively (*p = 0.60*). At 8 mm level, the loss was 0.52 ± 0.79 mm and 0.62 ± 0.57 mm, respectively (*p =* 0.56). No statistically significant difference was observed at all 5 different levels.

**Table 2:** Alveolar bone width changes and the percentages (Mean ± SD) at different vertical levels

Groups	AC	DBBM/CM	AC vs. DBBM/CM
Vertical levels	Bone width change (mm)	Bone width change (mm)	*P* value
**0 mm**	-3.71 ± 1.67	-3.53 ± 1.51	0.68
**2 mm**	-2.08 ± 1.17	-2.04 ± 1.34	0.91
**4 mm**	-1.21 ± 0.85	-1.33 ± 1.27	0.68
**6 mm**	-0.69 ± 0.64	-0.79 ± 0.87	0.60
**8 mm**	-0.52 ± 0.79	-0.62 ± 0.57	0.56
	**AC (%)**	**DBBM/CM (%)**	***P*** **value**
**0 mm**	-40.59 ± 15.29	-41.16 ± 18.44	0.90
**2 mm**	-20.75 ± 10.66	-22.14 ± 17.67	0.72
**4 mm**	-11.66 ± 7.97	-13.80 ± 15.36	0.51
**6 mm**	-6.56 ± 6.54	-8.09 ± 10.64	0.52
**8 mm**	-4.96 ± 6.19	-6.43 ± 7.23	0.41

### Vertical ridge height changes

**Fig. 5:**
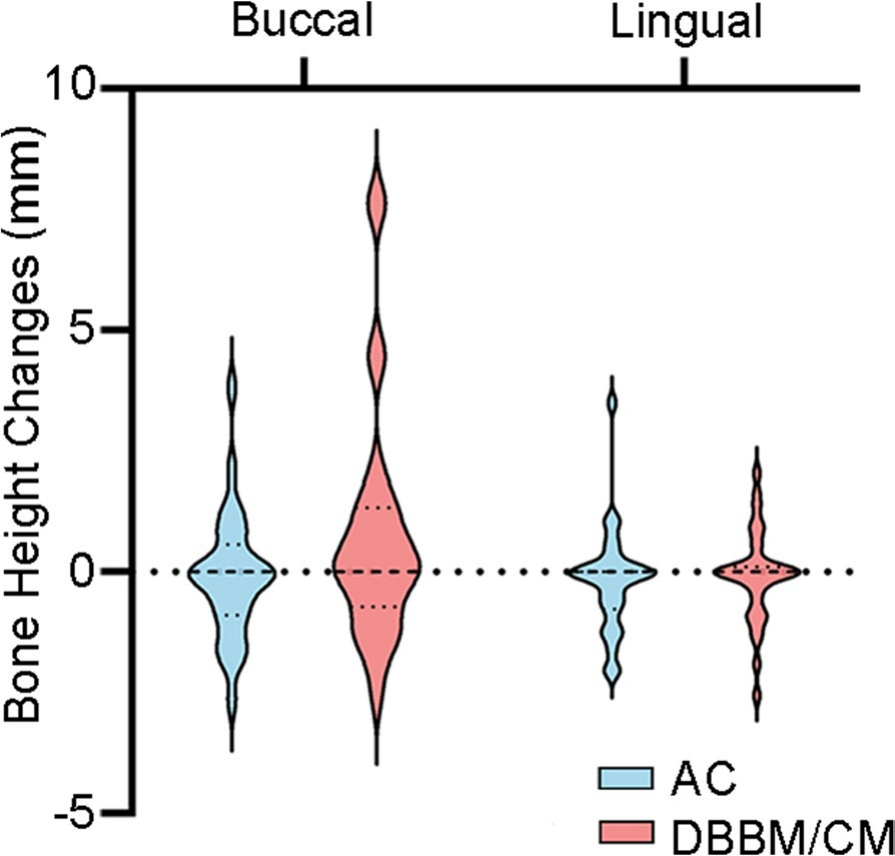
Measurement of buccal and lingual bone height changes at different levels in the AC group and DBBM/CM group

In AC group, the mean bone loss at the mid-buccal and mid-lingual was 0.10 ± 1.30 mm and 0.18 ± 1.08 mm, respectively. In contrast, in DBBM/CM group, it showed an opposite result of a gain of 0.77 ± 2.43 mm at the mid-buccal aspect. At mid-lingual aspect, it showed a loss of 0.10 ± 0.96 mm. The differences at both mid-buccal (*p* = 0.12) and mid-lingual (*p* = 0.91) between two groups were not statistically significant (Table 3; Fig. 5).

**Table 3:** Alveolar crest height changes (Mean ± SD) at buccal and lingual

	Alveolar crest height change	
	Buccal (mm)	Lingual (mm)
**AC (*N* = 28)**	-0.10 ± 1.30	-0.18 ± 1.08
**DBBM/CM (*N* = 30)**	0.77 ± 2.43	-0.10 ± 0.96
***P*** **value**	0.10	0.75

### Bone volume changes

**Fig. 6:**
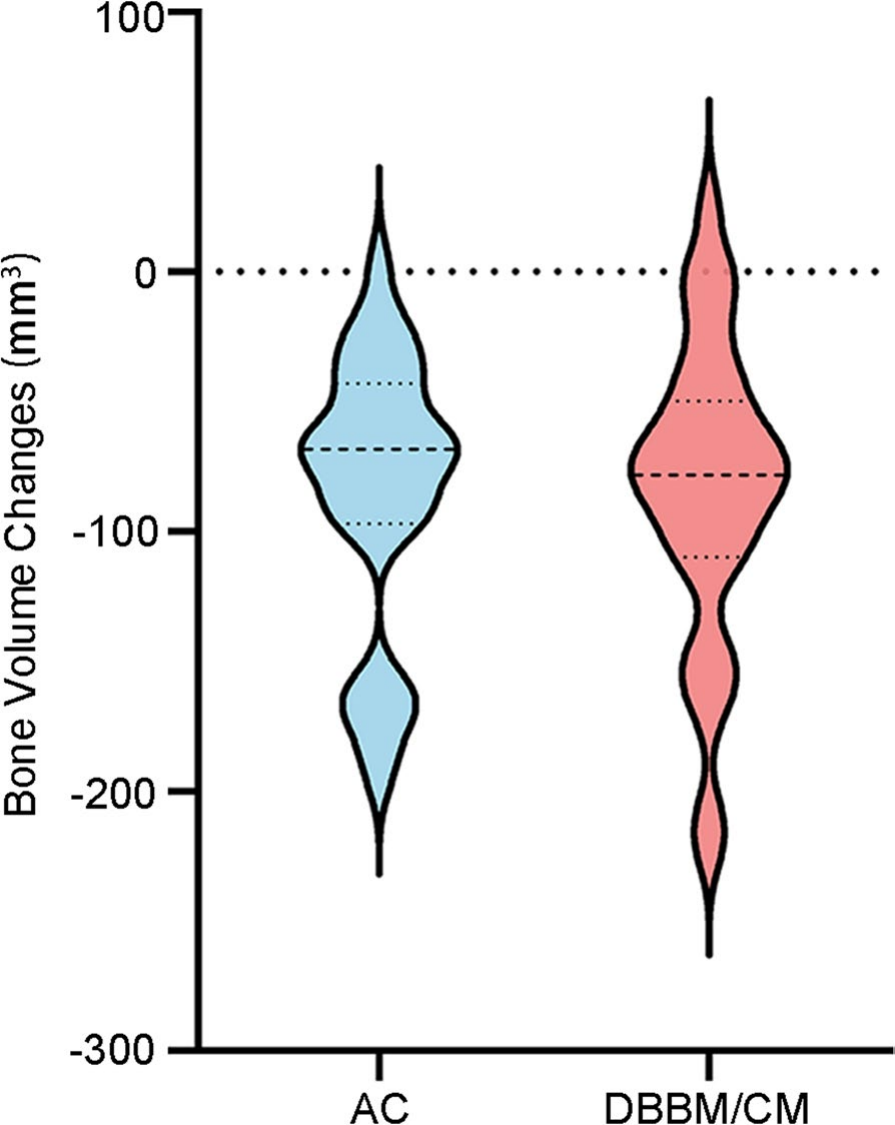
Measurements of bone volume changes in the AC group and DBBM/CM group

Volumetric outcomes were presented in Table 4; Fig. 6. Compared with preoperative, the mean volume reduction was 84.26 ± 52.93 mm^3^ in AC group and 84.27 ± 58.49mm^3^ in DBBM/CM group, which translated into a percentage volumetric reduction of 12.37%±6.09% and 14.54%±11.21%, respectively. The difference between two groups was not statistically significant (*p* = 0.99 and *p* = 0.36).

**Table 4:** Bone volume changes and the percentage (Mean ± SD) between the AC group and DBBM/CM group

	V_1_-V_0_ (mm^3^)	(V_1_-V_0_)/V_0_ (%)
**AC (*N* = 28)**	-84.26 ± 52.93	-12.37 ± 6.09
**DBBM/CM (*N*** **= 30)**	-84.27 ± 58.49	-14.54 ± 11.21
***P*** **value**	0.99	0.36

### Bone density measurement

**Fig. 7:**
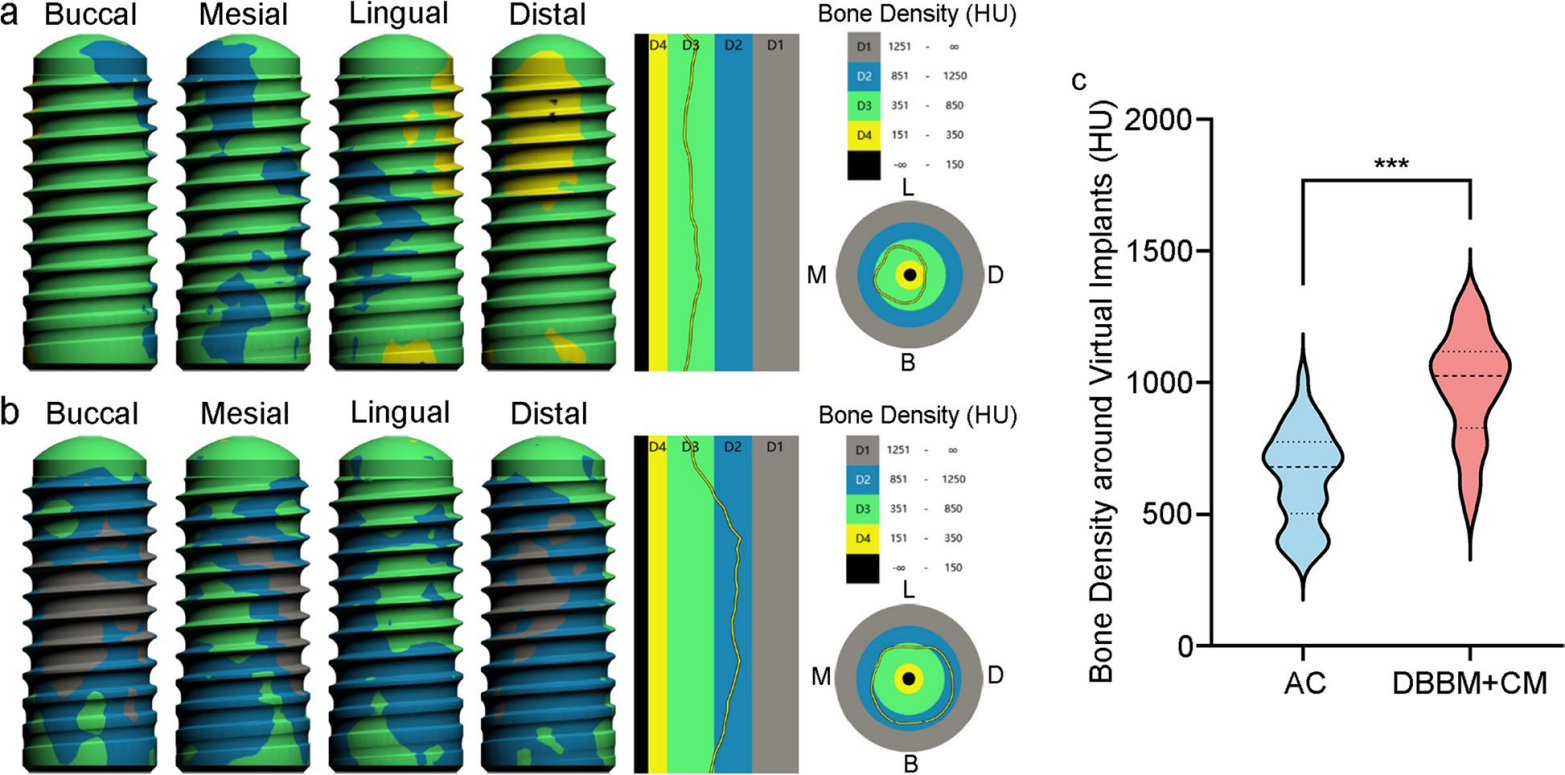
Bone density visualization and bone density measurement around virtually planned implant site. Typical bone density visualization in the AC group (**a**) and DBBM/CM group (**b**). Bone density measurement diagram (**c**)

From the bone density visualization diagram, the bone density around ideal implant site was dominated by type 3 bone in AC group (Fig. 7a). While most of the bone around the implant was type 2 bone in DBBM/CM group (Fig. 7b). As shown in Table 5; Fig. 7c. The average bone density around virtual implant of post-operative CBCT(T1) in AC group was 649.41 ± 184.71 HU, which was significantly lower than that in DBBM/CM group (985.23 ± 207.85 HU) (*p <* 0.001).

**Table 5:** Post-operative bone density around virtually planned implant site between the AC group and DBBM/CM group

	HUs around virtually planned implant site
**Atelocollagen (*N* = 28)**	649.41 ± 184.71
**DBBM/CM (*N*** **= 30)**	985.23 ± 207.85
***P*** **value**	< 0.001

## Discussion

To the best of our knowledge, this is the first human study to radiographically evaluate the effectiveness of AC in ARP. The present retrospective study was performed to compare the efficacy of AC vs. DBBM/CM in ARP through radiographic evaluation. The linear and volumetric analysis revealed no significantly different results between the two groups. However, the bone density in the AC group was significantly lower than DBBM/CM group (*p <*0.001), which was closer to type 3 bone according to the Lekholm & Zarb classification [28].

Natural healing may lead to horizontal bone loss of 29 ~ 63% and vertical bone loss of 11 ~ 22% after 6 months following extraction [29]. A previous meta-analysis found that reduction in width of the alveolar ridges was 3.87 mm, and radiographic crestal height reduction of the alveolar ridges was 1.53 mm [30]. Compared with the socket dimensional change in natural healing, both groups in present study seemed to have less horizontal bone loss and crestal height reduction. In present study, a decrease in radiographic bone width and volume in both groups was observed compared to the baseline. The currently available graft materials are unable to completely preserve the alveolar ridge after tooth extraction but can significantly reduce its resorption. These results of this study are consistent with the reports from the previous studies and systematic reviews [3, 5, 31, 32]. The present study showed that in the AC group and DBBM/CM group, horizontal ridge bone loss was 3.71 ± 1.67 mm (40.59%±15.29%) and 3.53 ± 1.51 mm (41.16%±18.44%) at the crest 0 mm of ridge, 2.08 ± 1.17 mm (20.75%±10.66%) and 2.04 ± 1.34 mm (22.14%±17.67%) at 2 mm apically, respectively. The horizontal width changes at 0 mm level in the DBBM/CM group in the present study seemed greater than a previous study which showed 1.84 ± 0.35 mm radiographic bone width loss [20]. The reason for this difference may be the choice of the linear measurement position. In the present study, postoperative imaging was used to determine the alveolar crest, which may be more coronal than that in the previous study. We measured at 5 different levels and a decreasing trend of the horizontal bone loss with an increasing distance to the alveolar crest in both groups was observed. One explanation for this outcome was that the crest of the buccal bone wall was comprised solely of the bundle which was resorbed and replaced with woven bone during the healing process of the extraction socket [1]. Another possible reason was that in some cases the socket bone wall was incomplete, where the ability of space maintenance was compromised, which may interfere the regeneration [33]. At all 5 different levels, the differences between the two groups were not statistically significant, which means AC may have a similar effect with DBBM/CM in limiting the horizontal dimension change of extracted socket.

In the present study, the buccal bone height in DBBM/CM group increased by 0.77 ± 2.43 mm. The result differed from the previous study, which showed a 0.31 and 0.32 mm decrease in buccal and lingual, respectively [18]. One explanation for the outcome was that in this study, there were several patients with damaged buccal bone plates included in this group (19/30), which resulted in a relatively low preoperative baseline level of buccal bone height. On the contrary, the results in the AC group showed bone height loss of 0.10 ± 1.30 mm at buccal and 0.18 ± 1.08 mm at lingual. The difference between the two groups may be that compared with DBBM, a low resorption rate material, the structure of collagen is looser, and the space maintenance ability is weaker. However, the difference between the two groups was not statistically significant.

In the present study, volumetric analysis was performed through a novel computer-based method that a VOI was selected from the three-dimensionally reconstructed and superimposed virtual models of the jaws [34]. Compared with traditional linear measures, this methodology allows a more precise, objective, and reproducible assessment of alveolar bone morphologic changes [35]. While the outcomes of bone volume reduction in the two groups were 84.26 ± 52.93 mm^3^(12.37%±6.09%) and 84.27 ± 58.49 mm^3^(14.54%±11.21%), respectively and no statistical significance was found.

Bone density analysis of post-operative imaging showed that the average bone density of the AC group was 649.41 ± 184.71 HU in the AC group and that of the DBBM/CM group was 985.23 ± 207.85 HU. The difference between the two groups was statistically significant. It was observed that HU values were not directly displayed from CBCT, representing a deviation from conventional CT. Nonetheless, previous studies indicate a strong correlation between the gray scale values of CBCT and HU values of CT [36–38]. Clinicians are therefore able to classify and assess bone density using CBCT. However, it should be noted that HU values derived from CBCT data and software reflect a ‘relative’ density, rather than actual density. In present study, the post-operative bone density of the AC group was lower and more similar to that of Quality 3 bone. Some previous histological or immunohistochemical studies may explain this result. A randomized controlled trial of ARP with collagen cone showed that the physiological healing process of the ARP site was similar to that of natural healing in histology, histochemistry, and immunohistochemistry, but it seemed to result in slightly higher values of von Willebrand factor, which may have a positive effect on vascularization [39]. On the contrary, in ARP with DBBM, the histomorphometric analysis revealed a high percentage of DBBM particles remaining on-site at 5 months, which may lead to a higher bone density [40]. In terms of clinical significance, the lower bone density in the AC group suggests that clinicians should pay more attention to the last drill and the bone extrusion in the process of implantation site preparation, to obtain higher primary stability [41].

Extensive clinical evidence has demonstrated the efficacy of collagen as a barrier covered the bone substitutes [19, 42] or an ingredient mixed with bone substitute materials in ARP [24, 43]. However, using collagen alone in ARP is still controversial in clinical practice, since it may have a negative effect on ARP [8]. In a randomized controlled study, collagen plug was used as a control group (group 1) to compare with three other different ARP techniques: group 2-socket grafting and polytetrafluoroethylene (PTFE) barrier; group 3-socket grafting, buccal overbuilding, and PTFE barrier; group 4-socket grafting, collagen barrier, and PTFE barrier. The results showed that the collagen plug was not inferior to the other three modalities based on keratinized mucosa and buccolingual ridge width changes and volumetric outcomes [35]. On the contrary, another study presented that the use of a Collacone (collagen sponge) does not enhance the bone and soft tissue healing outcome after extraction of an incisor in the maxilla compared with leaving the alveolus empty [44]. The compromised ARP outcome might result from the poor space maintenance ability and immunogenicity of collagen material. AC is a low-immunogenic collagen derivative with prolonged degradation time, which may overcome the drawbacks of normal collagen materials and have a better outcome in ARP. In the present study, the horizontal width changes at all 5 different levels and the volumetric outcomes were not statistically significant between the AC group and DBBM/CM group, which means AC might have a similar effect in limiting ridge dimensional change in ARP as DBBM/CM. The possible mechanism of AC preserving socket dimension is AC can help to stabilize the blood clot, which is critical for the regeneration process. Besides, AC does interfere healing process of the extraction socket due to its good biocompatibility and low immunogenicity, which have been confirmed in an animal study [17]. In addition, the prolonged degradation time of AC may enhance the space maintenance ability, which is important for bone regeneration. Nevertheless, an animal study has found that AC cannot help to preserve the socket dimension compared with natural healing [17]. The possible reason for this controversial outcome is the evaluation method in that animal study is histologic observation, which is hard to perform quantitative analysis. Also, the biological differences among different species may influence the outcome. It is worth noting that the use of AC alone in ARP has its unique advantages, such as convenience in clinical application and complete resorption of graft. In summary, AC could be an effective material in limiting the socket dimensional shrinkage after tooth extraction.

Regardless of these interesting findings, some limitations of the present study need to be addressed. First, the present study only evaluated the ARP outcome via radiographic data. We didn’t perform clinical measurements and histological analysis. It has been shown that radiographic measurements are inherently limited in terms of accuracy and underestimate alveolar bone dimensional loss compared with intra-operative measurements [45, 46]. In addition, the radiographic measurements of alveolar ridge dimensions may not reflect the true ridge dimensions since it is not possible to differentiate the new bone formation from the remaining graft particles on radiographs. Second, the ARP procedure is performed before implant installation, therefore implant-related outcomes as well as patient-reported outcomes are rarely reported. Third, the present study did not include a spontaneous healing group as a blank control. Finally, because of the limited number of cases and the limited follow-up time, the results presented in this article should be interpreted cautiously. Further studies should take these shortcomings into account. And randomized controlled trials with better design and longer follow-up time are needed to further verify the efficacy of AC in ARP.

## Conclusions

Within the limitations of this study, it can be concluded that AC can have a similar effect on limiting the dimensional alteration of the alveolar ridge when radiographically compared with DBBM/CM in ARP. However, neither of the techniques can completely preserve the alveolar ridge contour. Further studies with higher level evidence are needed to confirm our findings.

### Supplementary Information

**Additional file 1: Figure S1. **28 superimposed models of 28 patients used for measurement for region of interest (ROI) at AC group. The ROI in pre-operative models and post-operative models were indicated as yellow and green color, respectively. **Figure S2. **30 superimposed models of 30 patients used for measurement for region of interest (ROI) at DBBM/CM group. The ROI in pre-operative models and post-operative models were indicated as yellow and green color, respectively.

## Data Availability

The datasets used and analyzed during the current study are not publicly available due to limitations of ethical approval involving the patient data and anonymity but are available from the corresponding author on reasonable request.

## Abbreviations

AC: Atelocollagen
DBBM/CM: Deproteinized bovine bone minerals covered with a collagen membrane
ARP: Alveolar ridge preservation
CBCT: Cone-beam computed tomography
STROBE: Strengthening the Reporting of Observational Studies in Epidemiology
ROI: Region of interest
SD: Standard deviations
VOI: Volume of interest
PTFE: Polytetrafluoroethylene
