# Supplementary material for: Radiographic comparison of atelocollagen versus deproteinized bovine bone minerals covered with a collagen membrane in alveolar ridge preservation: a retrospective study
Source: BMC Oral Health. 2023 Nov 21;23:901. doi: 10.1186/s12903-023-03647-y (PMC10662564; doi:10.1186/s12903-023-03647-y)
Supplement: Supplementary file 1 — Additional file 1: Figure S1. 28 superimposed models of 28 patients used for measurement for region of interest (ROI) at AC group. The ROI in pre-operative models and post-operative models were indicated as yellow and green color, respectively. Figure S2. 30 superimposed models of 30 patients used for measurement for region of interest (ROI) at DBBM/CM group. The ROI in pre-operative models and post-operative models were indicated as yellow and green color, respectively. [file 12903_2023_3647_MOESM1_ESM.docx]

**Radiographic comparison of atelocollagen versus deproteinized bovine bone minerals covered with a collagen membrane in alveolar ridge preservation: a retrospective study**

Sha You^1,#^, Fan Yu^1,#^, Qihang Fan^1,2^, Ting Xia^1,2^, Liang Liang^3^, Qi Yan^1,2^, Hao Zeng^1,2,*^, and Bin Shi^1,2,*^

^1^ The State Key Laboratory Breeding Base of Basic Science of Stomatology (Hubei-MOST) & Key Laboratory of Oral Biomedicine Ministry of Education, School & Hospital of Stomatology, Wuhan University, China.

^2^ Department of Implantology, School & Hospital of Stomatology, Wuhan University, China.

^3^Department of Oral Implantology, Jianli Stomatology Hospital, Dongguan, PR China

^#^Sha You and Fan Yu contributed equally as first authors.

^*^Correspondence: hao.zeng@whu.edu.cn (H.Z) and shibin_dentist@whu.edu.cn (B.S)

**
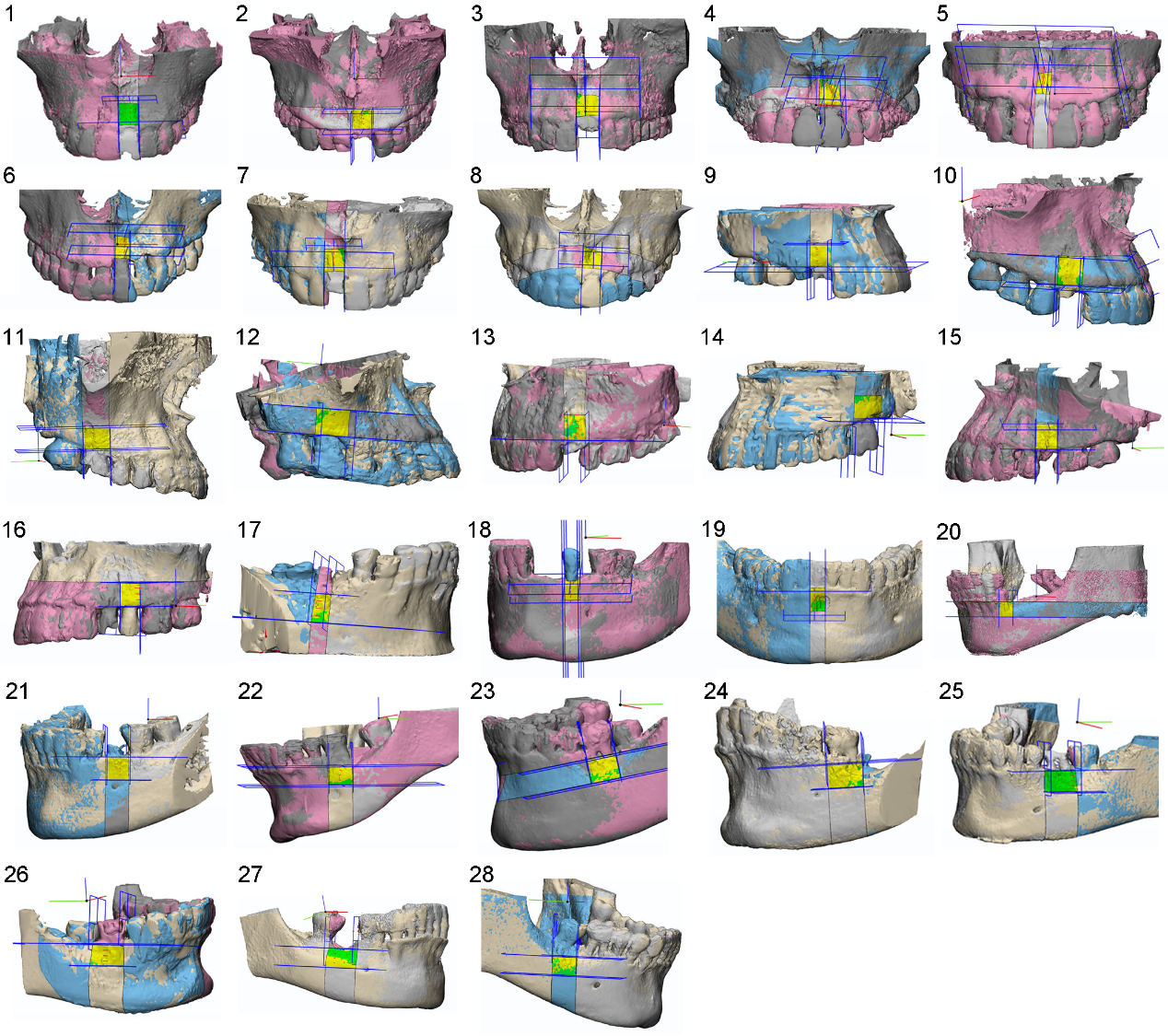
**

**Figure S1.** 28 superimposed models of 28 patients used for measurement for region of interest (ROI) at AC group. The ROI in pre-operative models and post-operative models were indicated as yellow and green color, respectively.


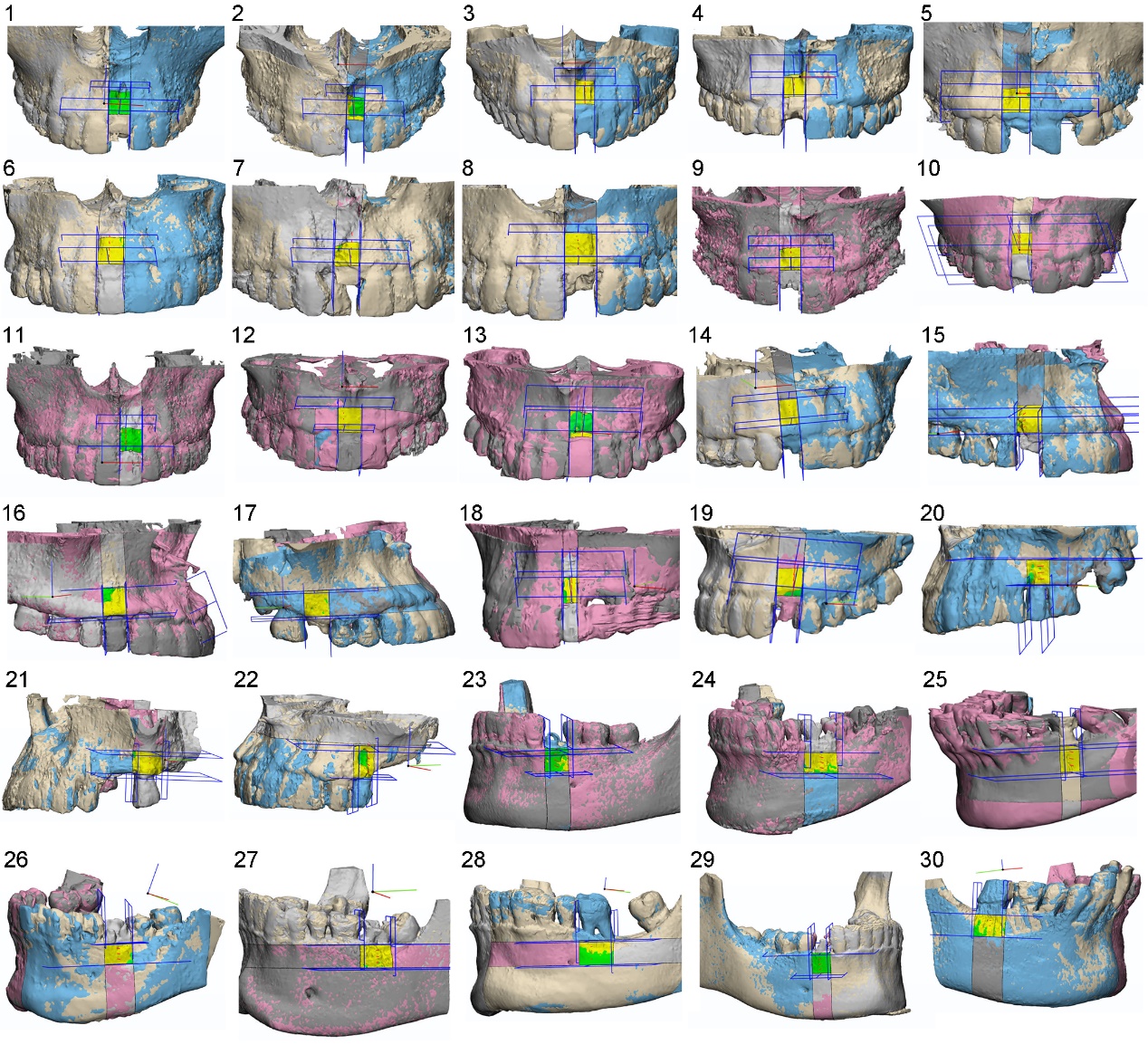


**Figure S2.** 30 superimposed models of 30 patients used for measurement for region of interest (ROI) at DBBM/CM group. The ROI in pre-operative models and post-operative models were indicated as yellow and green color, respectively.
